# Care of Children

**Published:** 1889-08

**Authors:** 


### AMERICAN JOURNAL

—OF—

### DENTAL SCIENCE.

VOL. XXIII. THIRD SERIES—AUGUST, 1889. No. 4.

ARTICLE I.

### CARE OF CHILDREN.

BAD AIR—CATCHING COLD—INDIGESTIBLE FOOD—POISONOUS FOOD AND DRINK—HOW PRECAUTION MAY SAVE A CHILD'S LIFE.

In order to guard against the summer complaint of infants the mother should know the causes of the disease. There are four sources from which it arises, which we may describe in order, one after the other:

*First.—Effect of Bad Air.*—The difference in the healthfulness of city and country depends largely on the fact that in the country the air is pure and free, while in the city the air is full of disease matter, which rises from cess-pools or closets or sewers or from filthy back-yards and alleys. Such poisonous air is not likely to produce indigestion directly, but it weakens the body so that it is not able to resist any disease which attacks it. This source of summer complaint cannot always be avoided, for the city

*Following is a series of articles, which "The Baltimore Sun" has had prepared by a well-known physician, on the ailments of babies in hot weather.

authorities neither keep the streets clean nor force the citizens to cleanse their premises. Much, however, can be done by the parents of the infant by cleaning and disinfecting their cesspools and gutters and drains, and by keeping all rubbish and decaying vegetables out of the house and cellar.

*Second.—Catching Cold.*—Every one knows that if a hot and perspiring infant is put into a cold draught of air it is likely to "catch cold." If it has not proper clothing on its stomach and belly, it will very likely get a diarrhœa. The way to prevent this is to clothe the infant so as to suit the weather and to keep it out of cool and damp currents of air. The child should wear flannel or merino or some other non-conducting cloth next its shin, and when this cannot be borne a bit of such cloth should be pinned over its stomach and belly, leaving the back and sides cool. If the weather changes two or three times a week the baby's clothing ought to be changed to suit. There is nothing more absurd than the old idea that the clothing should be changed for the season at such and such a day of the month.

*Third.—Indigestible Food.*—The only fit food for a baby before it begins to cut teeth is milk, with enough water to satisfy thirst between the nursings. A great deal of summer complaint is caused by giving the baby meats and vegetables and drinks which it cannot digest. There are very many mothers and fathers who bring up their little infants on "anything that they have on the table," beer, gin, cabbage, pork, unripe fruits, melons, &c. There are other parents who cannot refuse the little darling anything it cries for. To these classes it is useless to give instruction. They remind one of the opinion of the old negro concerning the education of certain young colored persons in the South: "Boss, I kin put more larrin into one of them niggers with a barrel stave than you kin with all the public schools in the State." There is no use instructing such parents, I say; they can only be taught by seeing their children die or grow up delicate. Even milk may at times disagree, but of this and the way to prepare milk I will speak in another article. It is the duty of every mother, if she can, to nurse her baby at the breast. If she cannot do this, cow's milk is the best and cheapest substitute. There is nothing that cheers the true physician's heart like having for the mother of his little patients a woman who gives to her little ones simple food which suits their tender years and *nothing else whatever to eat*, and who will treat as an enemy any kind-hearted neighbor or acquaintance who stuffs candies and indigestible foods into her baby's stomach.

*Fourth.—Poisonous Food and Drink.*—This is the chief cause of summer complaints. It is a fact that much of the water used throughout this country is poisonous. It contains the germs or seeds of disease. Modern science teaches that a great many diseases are caused by little germs so small that a million or so may be contained in a drop of water. If they are taken into the body in sufficient quantity for a long enough time they produce disease, each according to its nature. If the discharges of a person suffering with typhoid fever, for example, are thrown into a stream, or on the ground where they can be washed into a stream, they may cause typhoïd fever in nearly every one who drinks from the stream, because the typhoid germs in the discharges are carried by the water into the stomachs of the persons who drink it. We all know that if any one eats raw pork containing trichinæ he will suffer, perhaps die of disease which the little trichinæ produce. Our city hydrant water is generally wholesome, except when it has become stagnant in the ill-constructed water-pipes, but very many of the readers of *The Sun* drink water from wells or springs. In villages or cities all such water should be boiled for half an hour before it is given to infants, as it is likely to contain poisonous matter of various sorts, for long boiling kills or paralyzes most disease germs and drives out bad gases which may be in the water. Wherever there is a pure city water supply the use of water from wells or springs should be given up both for cooking and for drinking. The other way in which infants are most apt to take in disease-matter is in the milk which they drink. Milk from the mother's breast can hardly produce indigestion if the mother is healthy. Milk as it comes from a healthy cow at pasture is free from disease germs, though it may contain poisons from poisonous herbs eaten by the animal. Poisoning from herbs eaten by the animal must be extremely rare, so we need not fear it. The great trouble with the milk supplied to babies in our great cities is that disease germs get into it before it is delivered in the city, either from the air, or from the hands of the milkman, or from unclean vessels in which it is carried, or from impure water added to the milk by the milkman in his eagerness to have enough to go around, or used to wash the cans in. If no disease or fermentation germs get into the milk the heat of the sun or the shaking to which it is subjected will not hurt it. I will speak of this again when I come to treat of "Sterilized Milk." Cows which are kept in the city all the year, and especially those which are fed on slops and distillery waste products, are unsuitable for milch cows. Not having enough fresh air and exercise, they become unhealthy, and the milk which they give is unflt for use. A cow, to give good milk, must have good and wholesome food. Cattle often become consumptive if kept too closely housed and ill fed, and in many cases the milk of these cattle must contain the germs of consumption. The State authorities ought to have the power to forbid the sale of milk from cows which are not healthy or not properly fed and housed. The only way the people can avoid such milk is to buy milk only from country farmers, and to see that it is sweet-smelling and is not sour to the taste, and does not curdle nor settle on boiling.

*Review of the Four Causes.*—From this review of these four causes of summer complaint it will appear that it is possible for a very careful mother to prevent the disease in most cases. Every mother should understand the principles here explained, and do all she can to apply them in the care of her babies. Unfortunately the source of danger in many cases is the carelessness and uncleanness of others, who obey neither the laws of the State nor the teachings of their own conscience. Let us hope that as knowledge of the causes of disease increase, and as the administration of government becomes purer, a time will come when the wholesale destruction of infants seen each summer in our great cities shall be a thing of the past—when men shall wonder at the ignorance and the criminal neglect of known duty which filled the land at each returning hot season with suffering and lamentation. Well may we sympathize with the founder of a children's sanitarium, near this city, in his grief at the loss of his only children by the summer complaint, and in his determination "to devote part of the great wealth with which God had blessed him to the cure of little children afflicted with summer diseases, and to the study of the causes of this fatal affection and of the means by which it may be prevented."

### ARTICLE II.

The best infant food is the milk of a healthy mother. At the beginning of the child's life it is slightly aperient. This property is lost after the first few days or weeks. Mother's milk contains all the substances which are necessary to the building of the infant's body. In many cases, however, of feebleness or illness of the mother some other food is needed. Formerly it was the custom in this emergency to secure the services of a wet-nurse. The expense of hired nurses, the difficulty of obtaining healthy ones, the uncertainty as to whether they will be faithful, gentle and agreeable members of the family, have excited a prejudice against them, so that now wet-nursing is very seldom employed except in case of extreme illness of the infant. When very ill the partly-weaned infant almost always seeks the breast, and it is wise for mothers to continue to nurse their infants at intervals, even when they have to give them other food, especially during the first summer.

*The Milk of Animals.*—The milk of various animals has been used for the nourishment of infants, but practically none deserves mention save that of the cow. The milk of the ass or the goat may possibly be more like that of woman in some respects, but it is evident that for the present cow's milk will be generally used for this purpose, especially in our large cities, where artificial feeding is most frequently required. Cow's milk should be pure and sweet. When very rich it often disagrees with babies, the cream being, as it were, simply churned in the bowels and passed in little yellow cakes as large as a shirt button. When these little cakes are seen in the passages the milk should be diluted with water or the milk of another cow should be used. As long, however, as the milkman continues to mix the "milk of the cow with the iron tail" with the contents of his cans such dilution of the baby's food will not be needed. Many persons get the "milk of one cow" for their babies. Even if the milkman does furnish always the same cow's milk—which is very questionable—it is not at all certain that it is best for the baby to have it. If the cow chosen is an unhealthy one the baby will not do as well as with mixed milk. The milk should be slightly sweetened before it is given to the child and a little pinch of table salt should generally be added, as it improves digestion and prevents constipation. It is usually given mixed with water, in proportions varying according to the age of the child. Mothers boil the milk given to the baby in a tin, and give it as soon as it is cool enough; but there is a better method which I will presently explain, and every one knows that simply boiling the milk in this way will not keep the child safe from summer complaint. To understand the latest improvement in infant feeding one must know something of the germ theory of disease.

*The Germ Theory.*—By the aid of the best microscopes and with the help of various coloring matters students who work in great laboratories, such as those connected with our Johns Hopkins University, have discovered that there are very many different kinds of minute growths—" germs" they are called—so small that a million or more of them can live and move without crowding in a drop of water. They are of many shapes—some rod-shaped, some jointed, some spiral, some round. They are found everywhere—in the air, in the water, in the earth. When they have nothing to feed on they die or become sluggish; when they find a good pasture they grow and multiply at an astonishing rate. Freezing either kills them or prevents their activity; boiling, or heating to a temperature a little above boiling, will destroy them if it is kept up an hour or two. Very many changes with which we are all familiar are caused by these "germs." The souring of vinegar, the fermentation of beer and wines, the rising of bread are all produced by the action of little microscopic growths, which we know as "yeast," "mother," etc. Although these germs are so small, yet they cannot pass through dense cotton. If a liquid which can ferment is put into a flask with a tight stopper of cotton batting and boiled an hour or more it will never ferment until the cotton is removed, because all the germs inside the flask and in the cotton have been killed by the heat, and no other germs can get in through the cotton. The contents of such a flask are said to be "sterilized."

*Producing Poisons.*—Milk when it is just drawn from a healthy cow is free from germs; when it is exposed in the milk-pail to the air germs fall into it from the air and fermentation takes piece sooner or later. This fermentation is of different sorts, according to the nature of the germs which get in. One sort of germ will produce acid, turning the milk sour; other sorts produce substances which are very poisonous. When milk is kept cool—as in a dairy—the germs cannot grow well, and the milk remains a long time unchanged. When the milk is carried in cans for miles on the railroad and for miles through the streets on a hot day it quickly becomes sour, as the germs grow very rapidly, and even if it is not sour it may contain numbers of poison-producing germs. In a healthy child the juices of the stomach will destroy a reasonable number of germs, but the poison which has already been formed by the germs will cause sickness. To illustrate this fact, ice-cream, in which germs cannot grow, is sometimes very poisonous, poisons having been produced by the germs before it was frozen. In a child, who is weakened by hot weather, the juices of the stomach cannot destroy all the germs in the milk, but some of them pass into the bowels with the undigested milk, and there they grow and produce poisons which cause "summer complaint." Even if good milk is now given to the child it will be seized upon by the germs as soon as it reaches the bowels and fresh poisons will be produced from it. This is the cause of the serious vomiting, the diarrhœa and the fearful wasting of infants in hot weather.

The cure of these cases is often very difficult. As I have said, the use of pure milk will not cure the child. Certain medicines will help to prevent the poisonous changes or to destroy the germs. In addition to these means the baby must usually be taken to a cool country place, so that as it gains strength its stomach and bowels may be able to destroy the poison already in them. Even then it may be necessary to stop the use of milk for a time and to feed the baby entirely on beef teas, &c., which will not support the life of the germs, and which cannot be turned by them into poisons. After a time, when the germs are all dead or carried out of the body, pure milk may be given again.

It will be easy to see from these facts the great advantages of giving sterilized milk.

*To Prevent Sickness.*—"A stitch in time saves nine," and feeding the baby on sterilized milk when he is well saves the trouble of nursing him through sickness.

To sterilize milk put it when it first comes from the dairy into a number of nursing bottles, one for each nursing, filling them about three-quarters full and adding water, as much as is desired. Push a wad of cotton batting tightly into the mouth of each bottle. Then put the bottles into a pan of water and let the water boil for half an hour. Then set the bottles aside, and when one is needed take out the cotton and put on the nipple. Milk thus "sterilized" will keep all day without ice. If this method is followed milk which is free from poison when it is sterilized will remain free from poison until it is drank and will not disorder the child's bowels or stomach. I hope the time will come when the dairyman will "sterilize" all milk designed for infants' use immediately after it is drawn from the cow. If this is done it will be possible to deliver milk to customers in the hottest weather just as pure and fresh as when it left the cow, and the expense of ice will be saved. Milk so prepared at the dairy would bear transportation well in the large bottles—one for each family—in which it was sterilized, and if the mother on receiving it would sterilize it again in smaller nursing bottles, summer complaint would be unknown in her family. All drinking water used by the infant should be sterilized, although simple boiling is generally sufficient. Each bottle, when the milk is taken from it, should be at once washed with hot soapsuds and set aside full of water containing a little cooking soda. The nipple should be carefully washed with soapsuds and kept in a glass of water containing soda.

It is really sad to read in our daily papers articles in which the old methods, which have carried many an infant to an untimely grave, are still recommended by the writers, as if no better way were either known or needed.

About the "artificial infant foods" which are sold in the shops I need say but little. Many of them are very good in their way, but in at least one large sanitarium for children no use is found for them when the babies take sterilized milk. When good milk cannot be obtained, they are very useful.

Condensed milk is much used. It is prepared by evaporating the water from fresh milk. It is very serviceable when fresh milk is not to be had. We physicians meet now and then with infants fed on condensed milk who are slowly starving to death because the mother has not made it strong enough, having perhaps never taken the trouble to read the directions on the can in which it is held.

Beef tea and various meat broths and extracts are used sometimes for young babies, but they must be used carefully, as they may either irritate the stomach or cause diarrhœa. When the baby has cut his first teeth he may take beef juice or light broths daily, with soft pap of milk and bread or milk and crackers occasionally. As he grows older first finely-chopped, tender beef may be given, and then other articles may be added one by one as he is able to digest them.

